# 2009–2010 Influenza A(H1N1)-related critical illness among Aboriginal and non-Aboriginal Canadians

**DOI:** 10.1371/journal.pone.0184013

**Published:** 2017-10-19

**Authors:** James J. Jung, Ruxandra Pinto, Ryan Zarychanski, Deborah J. Cook, Philippe Jouvet, John C. Marshall, Anand Kumar, Jennifer Long, Rachel Rodin, Robert A. Fowler, On behalf of the Canadian Critical Care Trials Group H1N1 Collaborative

**Affiliations:** 1 University of Toronto, Faculty of Medicine. Department of Critical Care Medicine; Sunnybrook Hospital; Toronto, Ontario, Canada; 2 Department of Critical Care Medicine; Sunnybrook Hospital; Toronto, Ontario, Canada; 3 University of Manitoba, CancerCare Manitoba, Winnipeg MB Canada; 4 Departments of Clinical Epidemiology and Biostatistics and Medicine, McMaster University, Hamilton, Ontario, Canada; 5 Le Centre Hospitalier Universitaire Sainte-Justine, Université de Montréal, Montréal, Québec, Canada; 6 St. Michael's Hospital, Toronto, ON, Canada; 7 University of Manitoba Health Sciences Centre, Section of Critical Care Medicine, Winnipeg MB, Canada; 8 Department of Critical Care Medicine; Sunnybrook Hospital; Toronto, Ontario, Canada; 9 Public Health Agency of Canada, Ottawa Canada; 10 University of Toronto Departments of Medicine and Critical Care Medicine; Sunnybrook Hospital Toronto, Ontario, Canada; National Yang-Ming University, TAIWAN

## Abstract

**Background:**

Preliminary studies suggested that Aboriginal Canadians had disproportionately higher rates of infection, hospitalization, and critical illness due to pandemic Influenza A(H1N1)pdm09.

**Methods:**

We used a prospective cohort study of critically ill patients with laboratory confirmed or probable H1N1 infection in Canada between April 16 2009 and April 12 2010. Baseline characteristics, medical interventions, clinical course and outcomes were compared between Aboriginal and non-Aboriginal patients. The primary outcome was hospital mortality.

**Results:**

Of 647 critically ill adult patients with known ethnicity, 81 (12.5%) were Aboriginal, 566 (87.5%) were non-Aboriginal. Aboriginal patients were younger (mean [SD] age 40.7[13.7] v. 49.0[14.9] years, p < 0.001) and more frequently female (64.2% v. 51.1%, p = 0.027). Rates of any co-morbid illnesses (Aboriginal v. non-Aboriginal, 92.6% v. 91.0%, p = 0.63), time from symptom onset to hospital admission (median [interquartile range] 4 [2–7] v. 4 [2–7] days, p = 0.84), time to ICU admission (5 [3–8] v.5 [3–8] days, p = 0.91), and severity of illness (mean APACHE II score (19.9 [9.6] v. 21.1 [9.9], p = 0.33) were similar. A similar proportion of Aboriginal patients received antiviral medication before ICU admission than non-Aboriginal patients (91.4% v. 93.8%, p = 0.40). Among Aboriginal versus non-Aboriginal patients, the need for mechanical ventilation (93.8% v. 88.6%, p = 0.15), ventilator-free days (14 [3–23] v. 17 [0–24], p = 0.62), durations of stay in ICU (13[7-19.5] v. 11 [5–8] days, p = 0.05), hospital (19 [12.5-33.5] v. 18 [11-35] days, p = 0.63), and hospital mortality were similar (19.8% v. 22.6%, p = 0.56). In multiple logistic regression analyses, higher APACHE II score (1.06; 1.04-1.09, p<0.001) was independently associated with an increased risk of death; antiviral treatment with a lower risk of death (0.34; 0.15 – 0.78, p = 0.01). Ethnicity was not associated with mortality.

**Interpretation:**

During the 2009-2010 Influenza A (H1N1) pandemic, Aboriginal and non-Aboriginal Canadians with H1N1-related critical illness had a similar risk of death, after adjusting for potential confounding factors.

## Introduction

In the spring of 2009, the United States Centers for Disease Control and Prevention reported the occurrence of H1N1 Influenza A subtype in Southern California and Mexico.[1] Subsequently, H1N1 infection was reported across the globe and the World Health Organization declared a Phase 6 Influenza Pandemic, the first one of the 21^st^ century.[2] There have been several reports highlighting particular patient populations at potentially increased risk of severe illness.[3–5]

We previously reported baseline characteristics and outcomes of 215 critically ill patients with confirmed, probable or suspected H1N1 infection in the first pandemic wave in Canada.[3] Aboriginal Canadians comprise approximately 4% of the population, but accounted for 43 (25.6%) of those who were critically ill in this cohort.[3,6] In prior influenza pandemics, reports indicated that the North American Aboriginal population experienced disproportionately high morbidity and mortality.[7] During the 1918 H1N1 pandemic, the estimated mortality rate of Aboriginal communities was 3 to 9 times higher than that of other Canadian ethnic groups.[8,9] Speculation about underlying mechanisms of potentially increased illness burden have included biological and immune-mediated susceptibility, a differential rate of prior exposure and vaccination, particular co-morbid medical risk factors, differences in access to preventive health care and geo-social community closeness. Therefore, we performed an observational study to compare baseline characteristics, timeliness of healthcare access, illness severity, therapies delivered and clinical outcomes for critically ill Aboriginal and non-Aboriginal Canadians during the 2009 - 2010 Influenza A(H1N1) pandemic using predefined ethnicity categorizations.[10]

## Materials and methods

### Data sources

The Canadian Critical Care Trials Group designed a multicenter observational study (ICU-FLU) of critically ill patients infected with 2009 influenza A(H1N1) in April 2009. Data were collected retrospectively or prospectively on all patients with H1N1–related critical illness admitted in 51 Canadian hospitals between April 16 2009 and April 12 2010.[11] Details of data collection have been previously described.[4] The ICU-FLU database contains adult and pediatric critically ill patients admitted to participating hospitals in Canada with confirmed, probable, or suspected influenza including 2009-2010 H1N1 infection according to case definitions developed by the World Health Organization and the Public Health Agency of Canada.[12] Critically ill patients were defined as those admitted to an adult or pediatric ICU; those requiring mechanical ventilation (invasive or noninvasive); a fraction of inspired oxygen (FiO_2_) concentration greater than or equal to 60%; or intravenous infusion of inotropic or vasopressor medication.[3] The database includes demographic information such as age, sex, ethnicity, comorbidities, co-presenting illnesses, pregnancy status, symptoms at presentation, dates of symptom onset, ventilation initiation and liberation, severity of illness at presentation to ICU (Acute Physiology and Chronic Health Examination [APACHE] score II/III and Pediatric Risk of Mortality [PRISM] III score)[13,14], daily Sequential Organ Failure Assessment (SOFA),[15] and dates of hospital and ICU admission, discharge and death. Medications and ventilator therapies administered, nosocomial infections, and causes of death were also collected. Eligibility criteria and data were recorded by research coordinators or site investigators and validated for each centre. Centrally, data were checked for errors by manual inspection and electronic range limits.[16] Research ethics board (REB) approval was granted for this study by Sunnybrook Health Sciences Centre as the Central Coordinating Center (130-2009) and by each participating local REB thereafter. The requirement for informed consent was waived for this study as information was available from existing medical records. Patient records were anonymized and de-identified prior to analysis.

### Analyses

All analyses were performed to compare Aboriginal and non-Aboriginal critically ill patients for the period of April 16 2009 to April 12 2010. The primary outcome was mortality in hospital. Secondary outcomes were ventilator-free days (of first 28 days in ICU) and length of ICU and hospital stay in days.

Descriptive statistics included frequency analysis (percentages) for categorical variables and means (standard deviation [SD]), or medians (interquartile range [IQR]) for continuous variables. To test for differences in baseline characteristics between Aboriginal and non-Aboriginal critically ill patients, we used 2-sample t-tests for the continuous variables and χ^2^-test for the discrete variables. Outliers were temporarily censored until the data were confirmed. We used Kaplan-Meier curves to illustrate survival over the duration of follow up.

A number of pre-specified associations were investigated between patient level variables and the primary and secondary clinical outcomes. The accepted statistical practice of considering no more than one explanatory variable for every 5-10 patients who experience an event (response variable) of interest was employed in our model.[17] Univariate logistic regression was used to investigate the relationship between *a priori* explanatory variables and death in hospital, including ethnicity (Aboriginal/non-Aboriginal), age, sex, co-morbidities (including obesity, ever smoker status, chronic obstructive pulmonary disease, alcohol abuse, and diabetes, or *any* comorbidity), pregnancy or post-partum, time from symptom onset to hospitalization and ICU admission, severity of illness at presentation among adult critically ill patients (as evaluated by the APACHE II score), and treatment with antiviral medications prior to ICU admission or mechanical ventilation at admission. All pairwise associations between each explanatory variable were studied (Pearson’s r and Spearman’s r for nonparametric data) to ensure none were overly correlated (r ≥0.8). Ethnicity and each explanatory factor with p≤0.2 on univariate testing was singly entered into a multivariate model using forward selection, then confirmed using backward selection. Goodness-of-fit was examined for all multivariable models using the Hosmer-Lemeshow test. Linear regression employing forward and backward selection was used to relate ethnicity and other explanatory factors to the secondary outcomes (i.e., days free of mechanical ventilation of the first 28 days in ICU [18] duration of ICU and hospital stay). Confidence intervals and *p* values reported reflect a two-tailed α level of 0.05. Statistical analyses were conducted using SAS version 9.2 (SAS Institute, Cary, NC).

## Results

### Characteristics of study patients

Between April 16 2009 and April 12 2010, 754 critically ill patients were admitted to one of 51 participating ICUs met the study eligibility criterion of confirmed or probable Influenza A(H1N1)pdm09. Of these, 691 (91.6%) were adults and 62 were 18 years or younger; age was unavailable for one patient. Of the 666 adults with outcome data, ethnicity was available for 647 people: 81 (12.5%) classified as Aboriginal and 566 (87.5%) as Non-Aboriginal. Wave 1 included 42 (25.9%) people classified as Aboriginal, wave 2 included 39 (8.0%) people classified as Aboriginal (S1 Fig). All comparisons were between Aboriginal and all non-Aboriginal patients (n = 647) (Table 1).

**Table 1:** Demographic characteristics of critically Ill patients with confirmed or probable 2009/2010 influenza A(H1N1) infection, n = 647.

Ethnicity	No (%) of patients, n = 647
White/Caucasian	377 (58.3)
Aboriginal	81 (12.5)
First Nations	70 (10.8)
Inuit	8 (1.2)
Métis	3 (0.5)
South Asian (East Indian, Pakistani, Sri Lankan, Punjabi, Bangladeshi)	32 (5.0)
Asian (Chinese, Japanese, Vietnamese, Cambodian, Indonesian, Laotian, Korean, Filipino)	13 (2.0)
Black (African, Haitian, Jamaican, Somali)	9 (1.4)
Latin American (Mexican, Central/South American)	6 (0.9)
Arab/West Asian (Armenian Egyptian, Iranian, Lebanese, Moroccan)	3 (0.5)
Other	4 (0.6)
Unknown/Mixed	122 (18.9)

For the 81 Aboriginal v. 566 Non-Aboriginal patients, the mean [SD] age was 40.7 [13.7] v. 49.0 [14.9] years (p <0.001); 52 (64.2%) v. 289 (51.1%) (p = 0.027) were female, and 8 (15.4%) v. 19 (6.8%) (p = 0.0461) females were pregnant or post-partum. Almost half of the Aboriginal patients were admitted to ICU in Manitoba (37 (45.7%)) The Kaplan-Meier survival curves were similar (S2 Fig). At presentation, pre-existing co-morbidities were present in 75 (92.6%) Aboriginal v. 515 (91.0%) non-Aboriginal patients (p = 0.63). The 2 groups had similar presence of comorbidities, but a larger proportion of Aboriginal patients had alcohol abuse (25.9% v. 10.4%, p<0.001) than non-Aboriginal patients. Non-Aboriginal patients were more likely to have confirmed v. probable H1N1 status at presentation than did Aboriginal patients (Table 2).

**Table 2:** Baseline characteristics of critically Ill patients with confirmed or probable 2009/2010 influenza A(H1N1) infection by ethnicity, n = 647 ^a^.

Characteristic	Overall n = 647	Aboriginal n = 81	Non-Aboriginal n = 566	P value
Age in years (mean [SD])	48.0 (15.0)	40.7 (13.7)	49.0 (14.9)	<0.001
Female Gender (%)	341 (52.7)	52 (64.2)	289 (51.1)	0.027
Pregnant or Post-Partum (%)^b^	27 (7.9)	8 (15.4)	19 (6.6)	0.046
Any co-morbidity (%)	590 (91.2)	75 (92.6)	515 (91.0)	0.634
Ever Smoker (%)	268 (41.4)	45 (55.6)	223 (39.4)	0.0058
Diabetes (%)	171 (26.4)	19 (23.5)	152 (26.9)	0.52
Obesity (%)^c^	165 (25.5)	20 (24.7)	145 (25.6)	0.86
COPD (%)	112 (17.3)	12 (14.8)	100 (17.7)	0.53
Alcohol abuse (%)	80 (12.4)	21 (25.9)	59 (10.4)	<0.0001
APACHE II score (mean [SD])^e^	20.9 (9.9)	19.9 (9.6)	21.1 (9.9)	0.33
Eligibility Criteria				
Confirmed H1N1	555 (85.8)	61 (75.3)	494 (87.3)	0.0039
Probable H1N1	92 (14.2)	20 (24.7)	72 (12.7)	

Abbreviations: SD, Standard Deviation; COPD, Chronic Obstructive Pulmonary Disease; APACHE, Acute Physiology and Chronic Health Evaluation

^a^ Denominators vary among individual variables

^b^ Pregnancy count for female participants (n = 341).

^c^ Defined as a body mass index greater than 30 kg/m^2^.

^e^ APACHE II score available for 586 patients

### Course of illness and treatments received

The median time from initial symptoms to hospital admission was 4 [2–7] days for Aboriginal v. 4 [2–7] for non-Aboriginal patients (p = 0.84). The median time from initial symptoms to ICU admission was 5 [3–8] days for Aboriginal v. 5 [3–8] days for non-Aboriginal patients (p = 0.91) (Table 3). Illness severity at the onset of critical illness was similar (mean [SD] APACHE II score 19.9 [9.6] v. 21.1 [9.9], p = 0.33) (Table 2). Most Aboriginal and non-Aboriginal critically ill patients received mechanical (either invasive or non-invasive) ventilation (93.8% v. 88.6%, p = 0.18) (Table 3) and a similar proportion of Aboriginal patients (91.4% v. 93.8%, p = 0.40) received antiviral treatment before ICU admission.

**Table 3:** Clinical course of critically Ill patients with confirmed 2009/2010 influenza A(H1N1) infection by ethnicity, n = 647 ^a^.

Characteristic	Overall n = 647	Aboriginal n = 81	Non-Aboriginal n = 566	P value
Symptoms to Hospital Admission, days (median [IQR])	4 (2-7)	4 (2-7)	4 (2-7)	0.84
Symptoms to ICU Admission, days (median [IQR])	5 (3-8)	5(3-8)	5(3-8)	0.91
Mechanical Ventilation (%)^b^	573 (89.2)	76 (93.8)	497 (88.6)	0.15
Antiviral Treatment (%)^c^	605 (93.5)	74 (91.4)	531 (93.8)	0.40

Abbreviation: SD, Standard Deviation; ICU, Intensive Care Unit

^a^ Denominators vary among individual variables

^b^ Includes both invasive and non-invasive ventilation.

^c^ Includes use of oseltamivir or zanamivir up until ICU admission

### Outcomes

Mortality in hospital was 19.8% among Aboriginal patients and 22.6% among non-Aboriginal patients (p = 0.56). The median ventilation-free days (out of 28) was similar (14 [3–23] v. 17 [0–24] days, p = 0.62). The duration of stay in the ICU (median [IQR] 13 [7-19.5] v. 11 [5–8] days, p = 0.05) and hospital (19 [12.5-33.5] v. 18 [11-35] days, p = 0.63) were similar (Table 4).

**Table 4:** Outcomes of critically Ill patients with confirmed 2009/2010 influenza A(H1N1) infection by ethnicity, n = 64 ^a^.

Characteristic	Overall n = 647	Aboriginal n = 81	Non-Aboriginal n = 566	P value
Ventilation-free days (of 28) (median [IQR])	16.5 (0-24)	14 (3-23)	17 (0-24)	0.62
Length of ICU stay, days (median [IQR])	11 (6-19)	13 (7-19.5)	11 (5-18)	0.05
Length of Hospital Stay, days (median [IQR])	18 (11-34)	19 (12.5-33.5)	18 (11-35)	0.63
Deaths in hospital (%)	144 (22.3)	16 (19.8)	128 (22.6)	0.56

Abbreviation: SD, Standard Deviation; ICU, Intensive Care Unit

^a^ Denominators vary among individual variables

### Patient-level determinants of clinical outcomes

Increasing APACHE II score (odds ratio [OR] 1.06 per point, 95% CI [confidence interval] 1.04-1.09, p < 0.001) and neuraminidase inhibitor treatment (OR 0.34, 95% CI 0.15-0.78, p = 0.01) were significant independent predictors of in hospital mortality (Table 5). Aboriginal status was not significantly associated with ventilator-free days or duration of ICU or hospital stay.

**Table 5:** Univariate and multivariable logistic regression analysis modeling mortality in critically Ill patients with confirmed 2009/2010 influenza A(H1N1) infection, n = 647 ^a^.

	Unadjusted			Adjusted		
Variable	Odds Ratio	95% CI	P value	Odds Ratio	95% CI	P value
APACHE II Score	1.07	1.05,1.09	<0.001	1.06	1.04, 1.09	<0.001
Age, year	1.03	1.01,1.04	<0.001	1.01	1.00, 1.03	0.06
Sex, Female	0.87	0.60,1.26	0.46	0.82	0.54, 1.25	0.36
Ethnicity, Aboriginal	0.84	0.47,1.51	0.56	1.23	0.64, 2.37	0.54
Antiviral treatment	0.44	0.23, 0.84	0.01	0.34	0.15, 0.78	0.01
Obesity	1.51	1.01, 2.27	0.05	1.42	0.90, 2.23	0.13
Alcohol abuse	0.46	0.23, 0.92	0.03	0.50	0.24, 1.03	0.06
Pregnant^b^	0.28	0.06, 1.21	0.09	-	-	-
Any comorbidity	1.22	0.61, 2.42	0.57	-	-	-
COPD	1.13	0.70, 1.83	0.60	-	-	-
Symptoms to ICU, days	1.02	0.99, 1.06	0.26	-	-	-

Abbreviation: 95% CI, 95% Confidence Interval; COPD, Chronic Obstructive Pulmonary Disease; APACHE, Acute Physiology and Chronic Health Evaluation; ICU, Intensive Care Unit

^a^ Denominators vary among individual variables

^b^ Female subset, including pregnancy and post-partum state

## Discussion

After adjusting for differences in baseline characteristics, we did not find evidence of increased morbidity or mortality associated with Aboriginal status in the 2009-2010 H1N1 pandemic. Independent predictors of mortality included increasing severity of illness and lack of treatment with neuraminidase inhibitors consistent with other observational studies of patients with critical illness, acute lung injury, and H1N1-related illness.[3,4,13–15]

In 1918, the H1N1 pandemic mortality was potentially much higher (3 to 9 times) in Aboriginal communities in North America than in other ethnic communities.[9,19–25] In 1918, mortality within infected Alaskan and Labrador Inuit populations was reported as high as 30% to 90%.[19,23,24] In 2009-2010, many Aboriginal communities in Canada were far from tertiary medical care, such that worse outcomes of patients with H1N1-related critical illness may have been contributed to delayed management. We found that while duration from symptom onset to presentation was similar, fewer Aboriginal Canadians were treated with neuraminidase inhibitors before ICU admission.

The Aboriginal Canadian population is a mosaic of many communities and individuals, comprising First Nations, Inuit, and Métis. Approximately half of Aboriginal Canadians live in rural areas, on and off designated reserves.[25,26] Although our analysis is important in exploring certain potential risk factors for differential outcomes common among many Aboriginal Canadians and communities, we could not measure others, such as socioeconomic status, medical literacy and exact distance to tertiary medical care. Prior studies have documented that Aboriginal Canadians may suffer from decreased or delayed access to care and subsequently experience worse outcomes from AIDS, kidney disease and complications of diabetes, among conditions.[27–29] However, others have also found that while Aboriginal Canadians had a higher incidence of H1N1-related illness, particularly so in Manitoba during the pandemic’s first wave, the proportion of patients with severe outcomes was similar to the general population.[30]

Although certain jurisdictions had difficulty matching capacity for critical care with H1N1-related demand during short periods of time,[3] most regions were able to meet this demand with existing and augmented resources. If the 2009 H1N1 influenza virus had proved as lethal as in 1918, or if the number of cases had been greater, then the potential for differences in biologic susceptibility, access to prevention or to treatments may have led to worse clinical outcomes for Aboriginal Canadians. That mortality was higher in some international jurisdictions that experienced increased burden of illness in the face of limited capacity lends strength to this argument.[4]

Our study has a number of strengths. First, the ICU-FLU study represents a large national database of H1N1-related critical illness that contains detailed patient level information. Our data represent patients from all regions of Canada, enhancing the generalizability of our results. Our dataset contains the vast majority of all episodes of H1N1-related critical illness in Canada and it is unlikely that our findings represent selective case finding or would be altered by inclusion of subsequent patients.

Our study has important potential limitations. Valid characterization of ethnic status is challenging and it is possible that Aboriginal or other ethnic status was misclassified or unknown. However, trained clinical research coordinators collected ethnicity according to pre-defined categories suggested by Statistics Canada, using all information available to the clinical care team and hospital records.[31] As with any observational study, the associations that we found, cannot imply causation, and may be influenced by unmeasured or unrecognized confounding variables. We did not collect or adjust for other potentially important socio-economic variables or health literacy.

## Conclusions

We found that during the 2009-2010 Influenza A(H1N1) pandemic, despite a higher incidence of illness among Aboriginal populations, Aboriginal and non-Aboriginal Canadians with H1N1-related critical illness had a similar risk of death, after adjusting for potential confounding factors.

## Supporting information

S1 Fig Temporal distribution of critically Ill patients with confirmed or probable 2009/2010 influenza A(H1N1) infection by month during 2009-2010, n = 660. (TIFF)

S2 Fig Kaplan-Meier curve of survival among aboriginal and non-aboriginal canadians with confirmed or probable 2009/2010 influenza A(H1N1) infection by month during 2009-2010 n = 647. (TIFF)
