# Supplementary figures and images for: 2009–2010 Influenza A(H1N1)-related critical illness among Aboriginal and non-Aboriginal Canadians
Source: PLoS One. 2017 Oct 19;12(10):e0184013. doi: 10.1371/journal.pone.0184013 (PMC5648104; doi:10.1371/journal.pone.0184013)

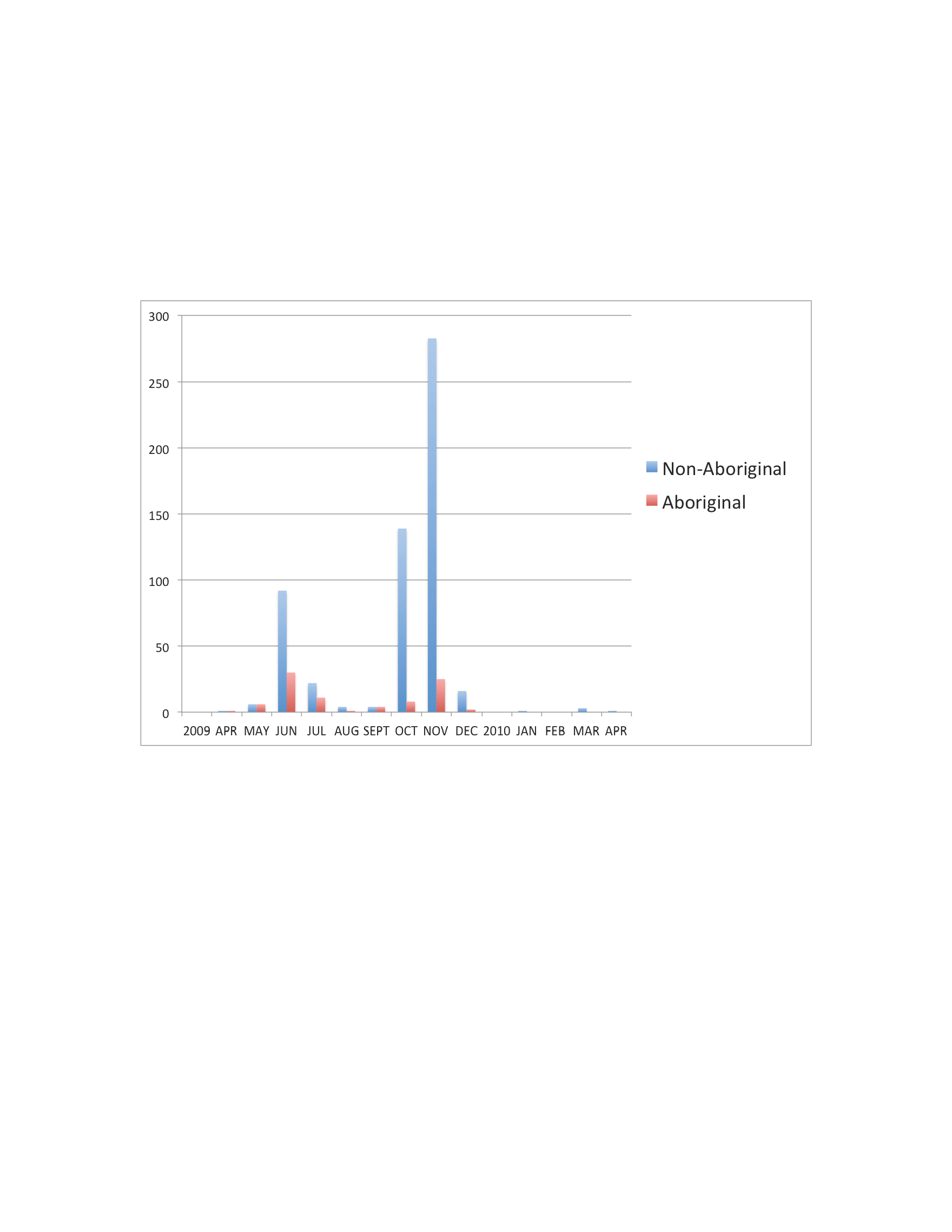

Supplement: S1 Fig — (TIFF) [file pone.0184013.s001.tiff]

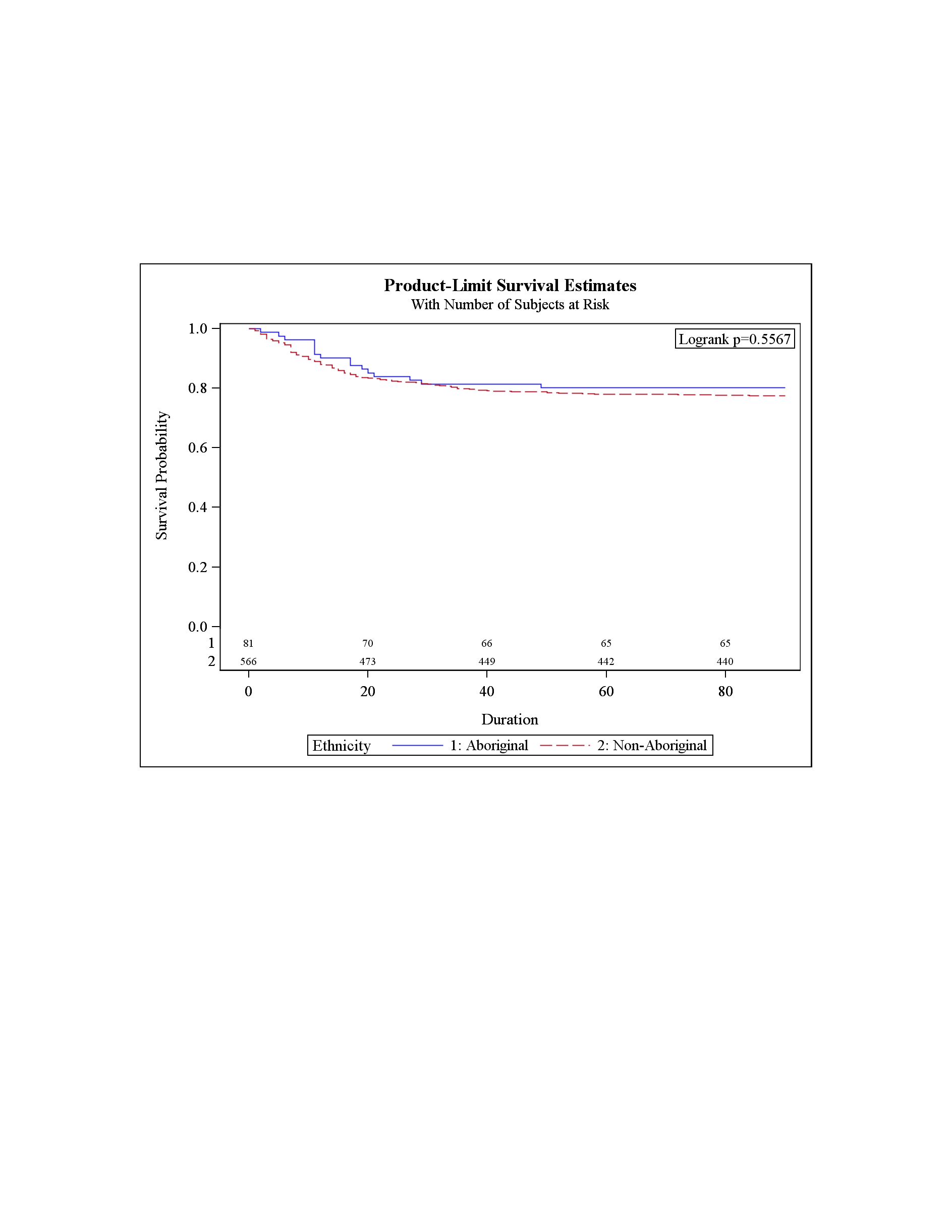

Supplement: S2 Fig — (TIFF) [file pone.0184013.s002.tiff]
